# Antitumor Activity of Alexidine Dihydrochloride Nanocarriers in Renal Cell Carcinoma

**DOI:** 10.1021/acs.molpharmaceut.5c00651

**Published:** 2025-09-02

**Authors:** Adan Sultan, Amani Zoabi, Anna Morshin, Ori Shalev, Philip Lazarovici, Katherine Margulis

**Affiliations:** † Drug Delivery & Mass Spectrometry Imaging Laboratory, The Institute for Drug Research, School of Pharmacy, Faculty of Medicine, 26742 The Hebrew University of Jerusalem, Jerusalem 9112001, Israel; ‡ The Harvey M. Krueger Family Center for Nanoscience and Nanotechnology, 26742 The Hebrew University of Jerusalem, Jerusalem 9190401, Israel; § Metabolomics Center, Core Research Facility, Faculty of Medicine, 26742 The Hebrew University of Jerusalem, Jerusalem 9112001, Israel; ∥ The Institute for Drug Research, School of Pharmacy, Faculty of Medicine, 26742 The Hebrew University of Jerusalem, Jerusalem 9112001, Israel

**Keywords:** phosphatidylglycerols, cardiolipin, protein-tyrosine phosphatase mitochondrial 1 enzyme (PTPMT1), desorption electrospray ionization mass spectrometry imaging (DESI−MSI)

## Abstract

Renal cell carcinomas (RCC) have recently been shown to exhibit a high abundance of phosphatidylglycerols, which are products of the protein-tyrosine phosphatase mitochondrial 1 enzyme (PTPMT1) and precursors of cardiolipins. Effective treatments for RCC are still in need. This study evaluates the therapeutic effect of PTPMT1 inhibition using the poorly water-soluble inhibitor alexidine dihydrochloride, which has not previously been proposed for RCC treatment. Considering that this inhibitor is poorly water-soluble and has inconsistent antitumor activity in its pure form due to solubility limits, we incorporated it in nanocarriers composed of phosphatidylcholine and cholesterol. These solvent-free nanocarriers had an average size of 66 nm, a drug loading capacity of 21%, an encapsulation efficiency of 99%, a positive surface charge, and excellent storage stability. We assessed their safety and efficacy in two human RCC cell lines, 786O and A498, alongside the human non-neoplastic kidney cell line HEK293 as a control. Results revealed a marked antitumor activity of the nanocarriers and selectivity toward highly metabolically active RCC cells. Thus, after only 24 h of treatment, a significant decrease in the viability of 786O cells was recorded, while A498 and control cells exhibited only minimal reductions in viability. Advanced mass spectrometry imaging (DESI–MSI) revealed that untreated 786O cells had significantly higher levels of phosphatidylglycerols, cardiolipins, and 4-hydroxynonenal glutathione compared to A498 and HEK293. Treatment with nanocarriers markedly impacted the levels of these metabolites in RCC cells. In conclusion, RCC tumors with upregulated phosphatidylglycerol metabolism may be particularly sensitive to PTPMT1 inhibition by nanocarriers.

## Graphical abstract

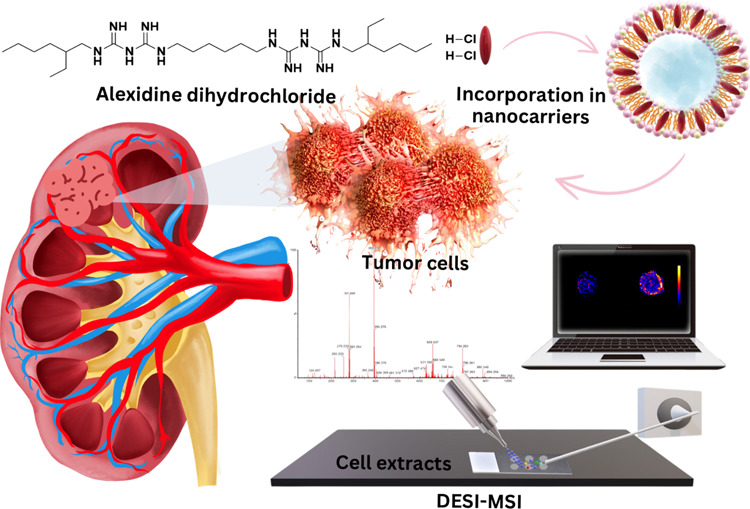

## 1 Introduction

Renal cell carcinoma (RCC) is a common urogenital cancer with a high mortality rate of 30–40%.^1^ RCC originates from the epithelial cells of the nephron;^2^ it grows and spreads quickly and often metastasizes to the liver, lungs, bones, and lymph nodes.^3^ Radical nephrectomy remains the main curative therapy for patients with localized RCC, but about 30% of these patients still develop metastases.^4^ Effective treatments for RCC are still very limited.^5^ Characterizing RCC oncogenic pathways and the related deranged metabolism can optimize the management of RCC and may offer novel therapeutic targets.^6^ One enzyme of interest is the protein-tyrosine phosphatase mitochondrial 1 (PTPMT1), which functions as the mammalian phosphatidylglycerol phosphate (PGP) lipid phosphatase, catalyzing the penultimate step of the cardiolipin biosynthetic pathway.^7,8^ Perturbations in cardiolipin (CL) homeostasis have previously been linked to apoptosis^9^ and can affect the tumorigenic potential of cells and the progression and aggressiveness of some types of tumors.^10^ Several studies have shown that in RCC tumors, there is a significant increase in phosphatidylglycerols (PGs), the direct products of PTPMT1, and precursors of CLs.^11,12^ Based on these data, we hypothesized that selective inhibition of PTPMT1 may present a possible metabolic treatment for RCC. In this study, we evaluated the effect of a specific PTPMT1 inhibitor, alexidine dihydrochloride, incorporated in nanocarriers, on the progression of RCC. Alexidine dihydrochloride is an alkyl bis­(biguanide) antiseptic used in mouthwashes to eliminate plaque-forming microorganisms and has broad-spectrum activity toward diverse fungal pathogens.^13^ Due to its low water solubility, alexidine dihydrochloride cannot be formulated in more than 0.05% (w/v) solution.^14,15^ Evidence shows that this compound may have antitumor activity due to its apoptosis-promoting action (by either PTPMT1 inhibition or disruption of mitochondrial respiration),^16^ but only when it is introduced using organic solvents, such as dimethyl sulfoxide. This was demonstrated in head and neck tumors, hepatocellular carcinoma, and, more recently, lung cancer and pancreatic carcinoma.^17–21^ Alexidine dihydrochloride has never been proposed for treating renal cell carcinoma. Moreover, the utilization of organic solvents may enhance the cell cycle arrest effect of alexidine dihydrochloride, but it also restricts its application *in vivo* and limits its potential as a clinically translatable therapeutic. Recent studies have addressed these limitations by developing structurally optimized alexidine analogs, demonstrating improved pharmacokinetics and *in vivo* efficacy, particularly in lung cancer models.^21^ These advancements underscore the need to enhance the drug-like properties of alexidine for broader therapeutic applications. To address these challenges, we explored an alternative strategy and incorporated alexidine dihydrochloride into water-dispersible nanocarriers. This approach aims to enhance solubility and bioavailability while preserving the integrity of alexidine’s chemical structure. The safety and efficacy of these nanocarriers were investigated using two different human renal cell carcinoma cell lines, 786O and A498, and the human embryonic kidney cell line HEK293 as a control (Figure 1). Clear cell renal cell carcinoma (ccRCC) is the most common type of RCC, with 786O and A498 being the most representative ccRCC cell cultures investigated.^22^ In this study, the levels of lipids and metabolites in the cells before and after the treatment with alexidine dihydrochloride-loaded nanocarriers were evaluated using mass spectrometry imaging, namely, desorption electrospray ionization mass spectrometry imaging (DESI–MSI).^23^ This imaging method enables direct analysis and mapping of molecules from the surface in ambient conditions at several micrometers depth, depending on imaging conditions.^23–28^ We demonstrate that inhibiting PTPMT1 with alexidine dihydrochloride incorporated in nanocarriers differentially affects the proliferation and viability of RCC cells, showing a stronger impact in tumors with elevated levels of phosphatidylglycerols and cardiolipins. Given that these nanocarriers exhibit favorable properties for efficient treatment, they may offer a novel, patient-specific approach for treating RCC.

**Figure 1:**
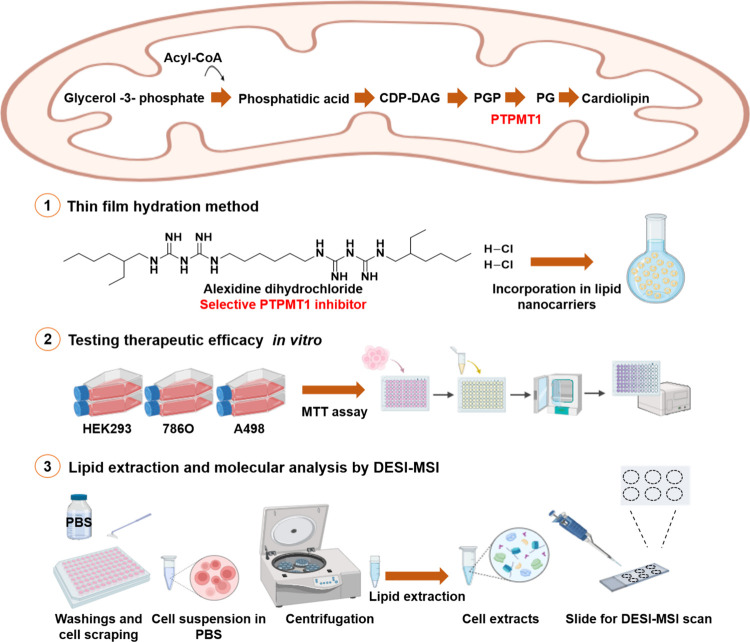
Schematic representation of the regulation of the cardiolipin biosynthesis pathway by PTPMT1 (upper panel) and the workflow of this study.

## 2 Materials and Methods

### 2.1 Cell Lines

HEK293 (an embryonic kidney cell line) and RCC cell lines 786O and A498 were obtained from ATCC (Manassas, VA, USA). All cell lines were cultured according to ATCC specifications. HEK293 and A498 cells were cultured in Dulbecoo’s Modified Eagle Medium (DMEM) supplemented with 10% (v/v) fetal bovine serum and antibiotics (100 mg/L penicillin and 100 mg/L streptomycin, Sartorius, Beit-Haemek, Israel).^29,30^ 786O cells were cultured in Roswell Park Memorial Institute medium (RPMI-1640) supplemented with 10% (v/v) fetal bovine serum and antibiotics (100 mg/L penicillin and 100 mg/L streptomycin).^31^ All experiments were conducted when cells were in the exponential growth phase.

### 2.2 Materials

Penicillin-streptomycin solution, fetal bovine serum (FBS), trypsin-EDTA 0.025%, and phosphate buffer saline (PBS) were purchased from Sartorius (Beit-Haemek, Israel); dimethyl sulfoxide was purchased from Thermo Scientific (Qiryat Shemona, Israel); thiazolyl blue tetrazolium (MTT reagent), trypan blue, and cholesterol were purchased from Sigma-Aldrich Merck (Jerusalem, Israel); alexidine dihydrochloride (1-[*N*′-[6-[[amino­[[*N*′(2-ethylhexyl)­carbamimidoyl]­amino]­methylidene]­amino]­hexyl]­carbamimidoyl]-2-(2-ethylhexyl)­guanidine dihydrochloride) was purchased from Apollo Scientific (Stockport, United Kingdom); and soybean phosphatidylcholine (Lipoid S100, phosphatidylcholine content ≥94.0%) was purchased from Lipoid (Ludwigshafen am Rhein, Germany).

### 2.3 Nanocarriers Preparation Using the Thin-Film Hydration Method

The thin-film hydration technique is commonly used to prepare MLVs (multilamellar vesicles).^32^ To ensure a homogeneous mixture, 200 mg of soybean phosphatidylcholine and 25 mg of cholesterol (286.66 g/mol) were dissolved in 20 mL of absolute ethanol and stirred for 20 min at room temperature. To remove the organic solvent, the ethanol was evaporated under a vacuum pump (Heidolph Instruments GmbH & Co, Schwabach, Germany) at a temperature of 40 °C. After removing the organic solvent, a homogeneous, dry, thin-lipid film was formed. The final stage was the hydration of the lipid film using 20 mL of double-distilled water. The hydration process was performed at 60 °C for 1 h while stirring to help detach the swelling lipid lamellae from the internal vessel surface to form MLVs (multilamellar vesicles). To downsize the nanocarriers, we performed finger sonication using Vibracell VCX750 (Sonics & Materials, Inc., CT, USA) for 15 min at 40% amplitude. Using the same method, we prepared nanocarriers with incorporated alexidine dihydrochloride by adding 60 mg of alexidine dihydrochloride to the ethanolic solution of soybean phosphatidylcholine and cholesterol prior to solvent evaporation. The final nanocarrier suspensions were stored at 4 °C for further characterization.

### 2.4 Characterization of Nanocarriers

#### 2.4.1 Size and ζ-Potential

The size and ζ-potential were measured by dynamic light scattering (DLS) using a Zetasizer Nano model ZEN3600 (Malvern Panalytical, Malvern, UK). All measurements were performed in triplicate. Three different batches of each system, nanocarriers with and without alexidine dihydrochloride, were evaluated. An intensity-weighted-*Z*-average diameter was reported for each measurement. For size measurements, the samples were diluted 30-fold with distilled water, and for ζ-potential, a 10 mM sodium chloride solution was used for the same dilution. The Zetasizer Nano instrument is equipped with a 633 nm laser, and light scattering is detected at 173° by backscattering technology. Zeta potential measurements were performed at 25 °C using a clear disposable zeta potential measurement cell using the same instrument.^33^

#### 2.4.2 Cryogenic Transmission Electron Microscopy (Cryo-TEM)

The morphology of the nanocarrier systems was examined using a cryo-transmission electron microscope, Tecnai G^2^ Spirit TWIN T12, (Thermo Fisher Scientific, Waltham, MA USA), having a resolution of 0.34 nm and equipped with a 4K FEI Eagle CCD camera.^34^

### 2.5 MTT Assay

The MTT assay was used to evaluate the *in vitro* therapeutic window of alexidine dihydrochloride nanocarriers and compare it with the one of alexidine dihydrochloride dissolved in 0.1% (v/v) DMSO.^35^ Cells were treated for up to 72 h with either 10 μM, 20 μM alexidine dihydrochloride, or 0.1% (v/v) DMSO that was used as a vehicle control. Alongside, alexidine dihydrochloride-loaded nanocarriers were also tested and compared to empty nanocarriers as a vehicle control, at concentrations ranging from 3.125 to 50 μM. The concentrations used in these experiments were selected based on previous studies that tested up to 20 μM alexidine in 0.1% (v/v) DMSO.^17^ Incorporating alexidine into nanocarriers allowed us to test higher concentrations, up to 50 μM, without the use of any organic solvents. Cells were cultured at 37 °C in a medium containing 10% (v/v) fetal bovine serum and antibiotics, with the medium changed every 48 h. Upon reaching confluency, the cells were detached with 0.025% trypsin, resuspended at 5 × 10^4^ cells/well in a 96-well plate, and incubated for 24 h to form a monolayer. Following this step, the medium was replaced and varying concentrations of alexidine dihydrochloride were added. For the nanocarrier experiments, a 4 mL 30 kDa centrifugal filter was used to isolate the nanocarriers with and without alexidine dihydrochloride for 90 min at 5000 rpm, followed by resuspension in cell culture medium to create dilutions ranging from 3.125 to 50 μM. Each concentration was added to wells in triplicates, with empty nanocarriers as vehicle control and nontreated cells as negative control. After incubation, 10 μL of MTT reagent (5 mg/mL) was added into each well, and cells were incubated for 3 h. Then, 300 μL of DMSO was used to dissolve the formed purple formazan crystals, and absorbance was measured at 540 nm (reference 650 nm) using a Tecan microplate reader (TECAN, SPECTRA Fluor PLUS, Salzburg, Austria). The viability of living cells was calculated as a percentage of the control.

### 2.6 Desorption Electrospray Ionization Mass Spectrometry Imaging (DESI–MSI) and Tandem MS

Cells were seeded in 6-well plates at a density of 5 × 10̂^5^ cells per well, with each well containing 2 mL of cell suspension. The plates were incubated for 24 h at 37 °C in a 5% CO_2_ atmosphere to establish a monolayer culture. Thereafter, the medium was removed and replaced with various concentrations of alexidine dihydrochloride-loaded nanocarriers suspended in either DMEM or RPMI-1640. Following another 24 h of incubation, the plates were placed on ice, and the wells were washed twice with 2 mL of cold PBS. Cells were then harvested using Costar cell scrapers, transferred to 1.5 mL Eppendorf tubes, and centrifuged at 1000 rpm for 10 min at 4 °C. After centrifugation with an SL 16R centrifuge (Thermo Fisher Scientific, Waltham, MA, USA), the PBS was removed by vacuum aspiration. The cell pellet was then dissolved in 1 mL of absolute methanol, vortexed for 1 min at maximal speed, and sonicated in a bath sonicator (MRC Ltd., Holon, Israel) at room temperature for 30 s. The Eppendorf tubes were centrifuged at 15,000 rpm at 4 °C for 10 min. At the final stage, 500 μL of lipid extraction was removed to a new Eppendorf tube, 500 μL of sterile water was added, and the solution was vortexed for 1 min and stored at −80 °C for further analysis. For DESI–MSI analysis, 0.4 μL of cell extracts were applied on a glass microscope slide. These extracts were obtained from cells either treated with 12.5 μM alexidine dihydrochloride-loaded nanocarriers and incubated for 24 h or from untreated control cells and were applied to the same slide. This approach ensured that all samples were analyzed under identical instrumental and matrix conditions, thereby minimizing variability in ionization efficiency.

DESI–MSI was performed on a Xevo G2-Si mass spectrometer equipped with a DESI-XS stage (Waters, Milford, MA, USA).^26^ After optimization of spray solvent composition, source parameters, and mass spectrometry parameters, the chosen parameters were as follows: (1) a negative ion mode was used; (2) the spray solvent was methanol (MeOH/DDW, 98:2 v/v); (3) the spray flow rate was 2 μL/min; (4) the spray voltage was set to 0.7 kV; (5) the sampling cone voltage was set to 40 V; (6) the temperature of the heated transfer line was 450 °C; (7) and the imaging step size was 70 μm. Region of interest (ROI) analyses were performed on the MSI ion images of treated and untreated cells using high-definition imaging (HDI) software, version 6.2. Average mass spectra were obtained by integration over the entire area of each cell extract. The intensities of relevant metabolites were compared between treated and untreated cells. The chemical identities of the ions were confirmed using tandem MS in negative mode with a Q-Exactive Plus Orbitrap (Thermo Fisher Scientific, Waltham, MA, USA) mass spectrometer in a higher-energy collisional dissociation (HCD) mode. We used selected-ion monitoring (SIM) analysis to optimize the signal intensity for each peak of interest. FreeStyle software (Thermo Fisher Scientific, Waltham, MA, USA 1.8 SPF2 QF1, version 1.8.65.0) was used for quality control and visualization throughout the data evaluation process.

### 2.7 Cell Counting

The cells were counted using a Luna- II automated cell counter (Logos Biosystems, VA, USA). Cells were dispersed using 0.025% Trypsin-EDTA and gentle pipetting. Afterward, each cell suspension was mixed with 10 μL of 0.4% trypan blue at a ratio of 1:1. Then, it was homogenized for 10 s using a 10 μL micropipette. Next, 10 μL of mixed cells and trypan blue was transferred into the counting slide. Finally, the slide was inserted into the device, and the monitor displayed the number of live and dead cells per milliliter and the viability of each cell suspension after a few seconds.

### 2.8 Ultraviolet–Visible Spectroscopy, Encapsulation Efficiency, and Loading Capacity

The nanocarriers were filtrated using a 500 μL 30 kDa Eppendorf filter, and 500 μL of the nanocarrier suspension in water was centrifuged at 5000 rpm for 5 min. Afterward, 100 μL of the nanocarrier suspension was diluted 20-fold with absolute ethanol. The resulting solution was analyzed for alexidine dihydrochloride content (UV absorbance at λ = 237 nm) using a UV spectrophotometer (Amersham Biosciences, Amersham, UK). Before this experiment, a calibration curve for alexidine dihydrochloride UV absorbance was prepared to calculate the encapsulation efficiency. Encapsulation efficiency (EE %) was calculated by (total drug added – free nonentrapped drug) divided by the total drug added. Loading capacity is the amount of drug loaded per unit weight of the nanocarriers, which was calculated by the amount of the total entrapped drug divided by the total nanocarrier weight.

### 2.9 Statistical Analysis

The data were analyzed by PRISM software. One- and two-way analysis of variance (ANOVA) with the post-Tukey test was used to determine significant differences between the groups. The level of significance was set at *p*-value <0.05.

## 3 Results and Discussion

### 3.1 Synthesis and Physicochemical Characterizations of Alexidine Dihydrochloride Nanocarriers

Alexidine dihydrochloride has negligible aqueous solubility but is freely soluble in ethanol. Therefore, a thin-film hydration technique was selected to incorporate this compound into nanocarriers containing soybean phosphatidylcholine and cholesterol (Figure 2A). The resultant nanocarriers were freely dispersible in water upon reconstitution. Unloaded and alexidine dihydrochloride-loaded nanocarriers were prepared and characterized (Figure 2). The nanocarrier size was 87 ± 1 nm (PDI 0.37 ± 0.004) before the incorporation of the drug and decreased to an average of 66 ± 0 nm (PDI of 0.24 ± 0.009) upon incorporation (Figure 2B). ζ-potential was −6.6 ± 0.6 mV before the incorporation of the drug and grew significantly after the incorporation, measuring +39.1 ± 1.3 mV. Based on these results, we infer that the cationic amphiphilic alexidine dihydrochloride is localized within the phospholipid bilayer, increasing the ζ-potential and causing the overall decrease in nanocarrier size probably due to its effect on the bilayer curvature. Previous studies have shown that incorporating amphiphilic drugs into the bilayer changes the geometric structure of nanocarriers, impacting their size, surface bilayer curvature, and rigidity.^36^ The encapsulation efficiency of alexidine dihydrochloride was determined with UV–vis by measuring absorbance at 237 nm and calculating the percentage of alexidine dihydrochloride entrapped in the nanocarriers (Figure 2C). A calibration curve for alexidine dihydrochloride absorbance in absolute ethanol that is directly proportional to concentration was prepared for this purpose (Figure S1). The calculated encapsulation efficiency was 99%, and the loading capacity was 21%. To confirm the size of the nanocarriers and characterize their morphology, cryo-TEM evaluation of the alexidine dihydrochloride-loaded and unloaded nanocarriers was performed. The images reveal a diversity of sizes and lamellar properties within each group. Nanocarriers with incorporated alexidine dihydrochloride were smaller, had a more homogeneous lamellar substructure, and could be defined as unilamellar vesicles (Figure 2D). By contrast, unloaded nanocarriers were larger and showed a heterogenic lamellar structure and a higher content of multilamellar vesicles (Figure 2E). Next, to assess the stability of the systems, the size of loaded and unloaded nanocarriers was measured after six months of storage. The size of the loaded nanocarriers was 68 ± 1 nm (PDI of 0.24 ± 0.003, roughly 3% size increase) (Figure S2A), and the size of the unloaded nanocarriers was 93 ± 1 nm (PDI of 0.4 ± 0.019, roughly 7% size increase) upon storage (Figure S2B). This indicates that cationic amphiphilic alexidine dihydrochloride, located within the phospholipid bilayers, improves the long-term stability of the system. Overall, the resultant nanocarriers demonstrate high aqueous dispersibility, a size ideal for leveraging the enhanced permeability and retention (EPR) effect, and a positive surface charge that can potentially promote efficient transvascular transport.^37^ These characteristics, combined with high drug loading, encapsulation efficiency, and storage stability, make these nanocarriers promising candidates for *in vivo* RCC treatment.

**Figure 2:**
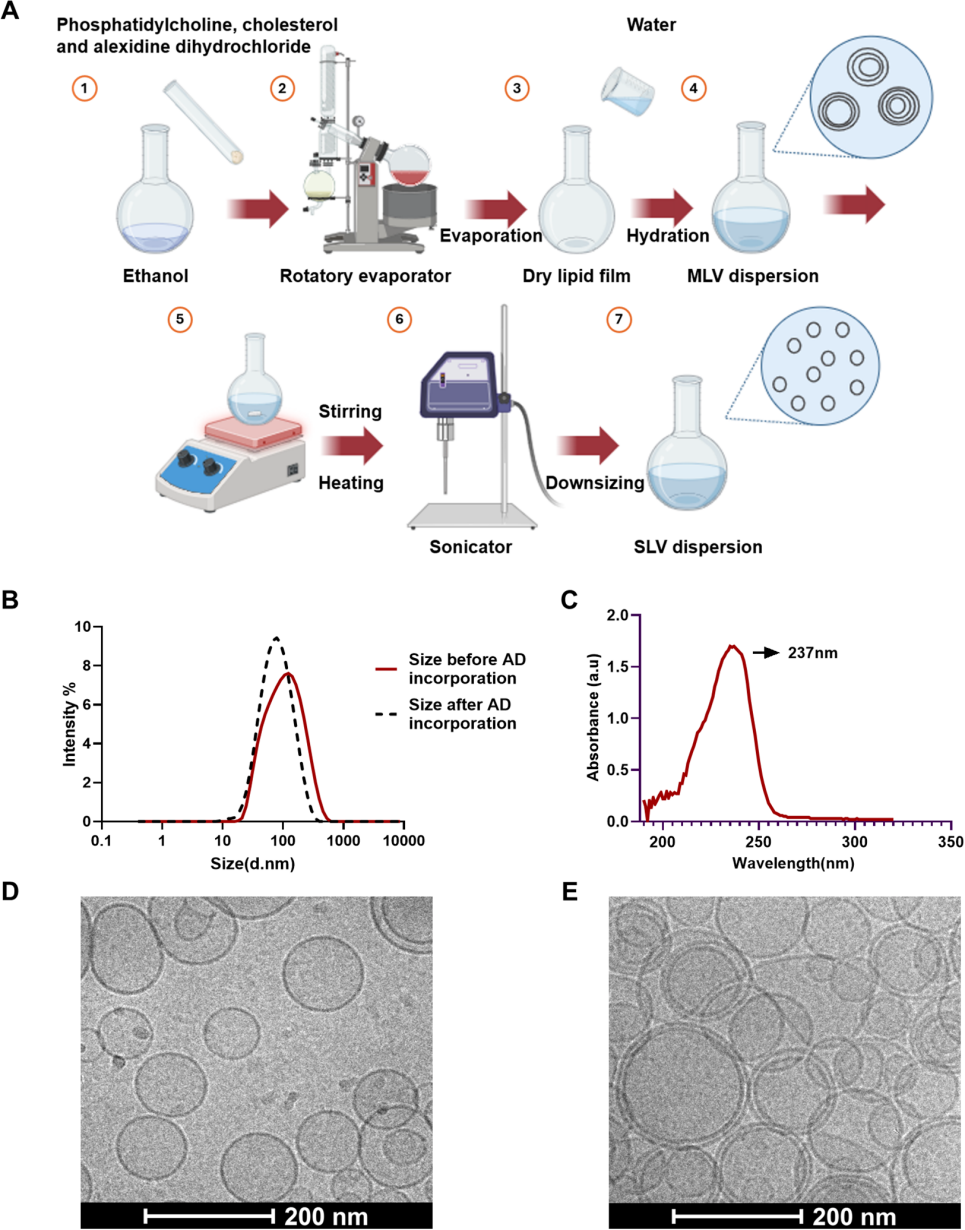
(A) Schematic representation of the main stages of the thin-film hydration method used for nanocarrier preparation (please refer to the Methods section for details). (B) Representative size measurement of nanocarriers before (solid graph) and after (dashed graph) the incorporation of alexidine dihydrochloride; *d*. nm: diameter in nm. (C) UV absorbance spectrum of alexidine dihydrochloride incorporated in the nanocarriers. (D) Cryo-TEM image of nanocarriers loaded with alexidine dihydrochloride in double-distilled water. (E) Cryo-TEM image of unloaded nanocarriers in double-distilled water.

### 3.2 Effect of Alexidine Dihydrochloride-Loaded Nanocarriers at Low Concentrations on Cell Viability

The impact of alexidine dihydrochloride on cell viability was evaluated by using the MTT assay. Alexidine dihydrochloride dissolved in DMSO exhibited inconsistent cytotoxicity, likely due to its poor aqueous solubility, highlighting the need for solvent-free nanocarriers (Figure S3). Indeed, alexidine dihydrochloride-loaded nanocarriers demonstrated more consistent antitumor activity, significantly reducing cell viability in both RCC (786O) and control (HEK293) cells at all tested concentrations and time points (Figure S4). To minimize toxicity to control cells, the drug concentration was subsequently reduced.

To evaluate the cytotoxic effect of alexidine dihydrochloride-loaded nanocarriers at lower concentrations, RCC cell lines 786O and A498 and the control cells HEK293 were incubated with alexidine dihydrochloride-loaded nanocarriers at a 3.125–12.5 μM drug concentration range. Cell viability was determined by the MTT assay over 72 h. The unloaded nanocarriers were tested as a vehicle control, and untreated cells were tested as a negative control. The cytotoxicity assay results are presented in Figure 3. A significant dose-dependent decrease in cell viability was observed in 786O cells at all concentrations and time points. In contrast, HEK293 cells showed no significant decrease in viability after 24 h at any concentration, while A498 cells only exhibited a decrease at the highest concentration (12.5 μM). HEK293 cells responded to treatment only after 48 and 72 h with higher concentrations of alexidine dihydrochloride (6.25 and 12.5 μM). Similarly, A498 cells showed a response to treatment only after 48 and 72 h.

**Figure 3:**
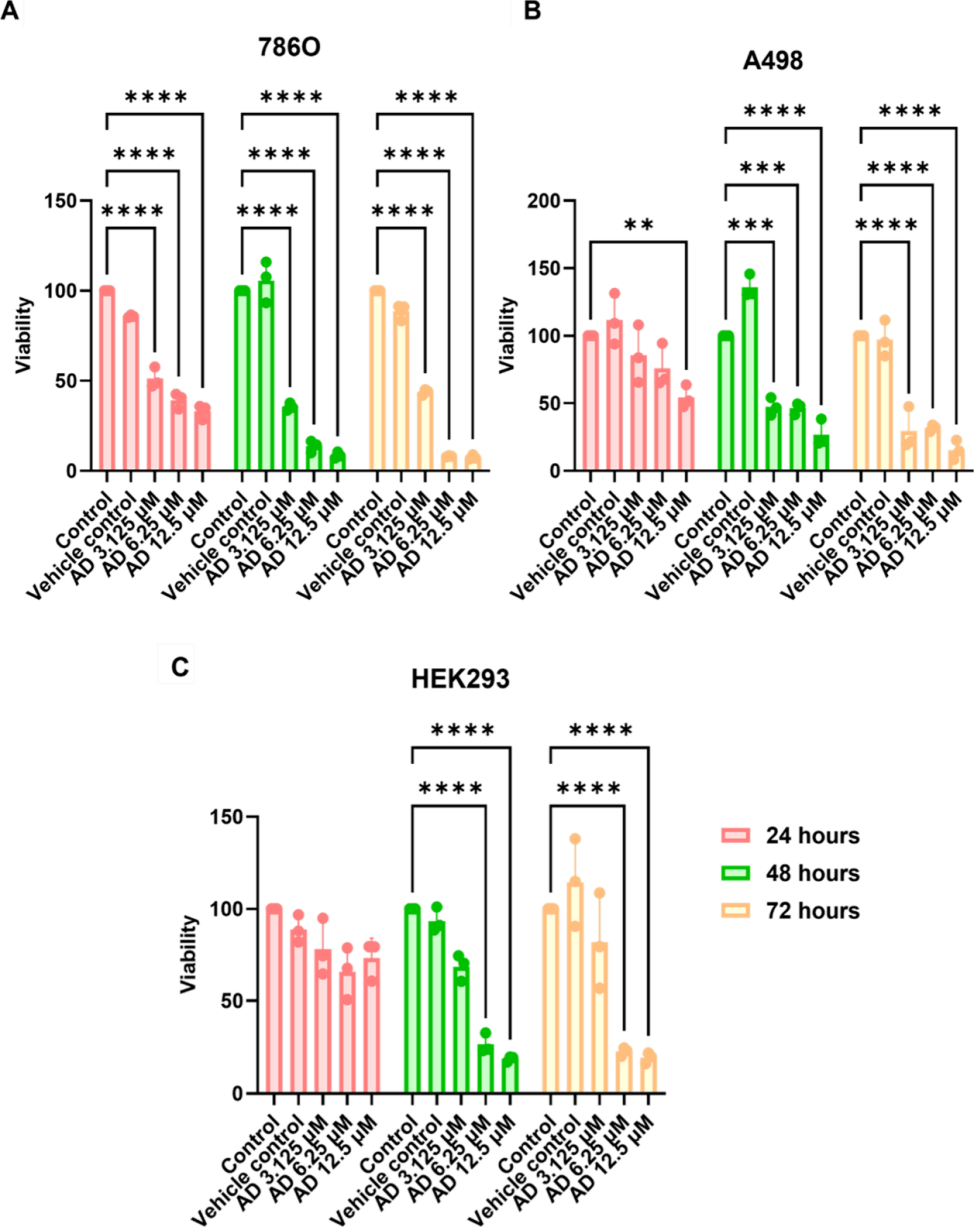
Cell viability analysis using the MTT assay. (A) 786O cell viability. (B) A498 cell viability. (C) HEK293 cell viability. Control = untreated cells; vehicle control = cells treated with empty nanocarriers; AD 3.125 μM = cells treated with 3.125 μM alexidine dihydrochloride incorporated in nanocarriers; AD 6.25 μM = cells treated with 6.25 μM alexidine dihydrochloride incorporated in nanocarriers; AD 12.5 μM = cells treated with 12.5 μM alexidine dihydrochloride incorporated in nanocarriers. The data shown represent three separate experiments, and values are given as mean ± SD. Statistical analysis was performed by two-way analysis of variance with all pairwise multiple comparison procedures done by the Tukey test. Symbols: **P ≤ 0.01, ***P ≤ 0.001, and ****P ≤ 0.0001.

To further explore these differences in response, we employed a novel mass spectrometry imaging method—desorption electrospray ionization mass spectrometry imaging (DESI–MSI)—to detect metabolites extracted from untreated and alexidine dihydrochloride-loaded nanocarrier-treated 786O and A498 RCC cells and compare them to control HEK293 cells.

### 3.3 Alexidine Dihydrochloride in Nanocarriers Inhibited the Synthesis of Phosphatidylglycerols and Other Phospholipids in RCC

We performed DESI–MSI on treated and untreated cells to evaluate the effect of alexidine dihydrochloride on metabolite levels. Overall, higher relative intensities of lipid ions were observed in untreated tumor cells 786O and A498 compared with those in untreated HEK293. The highest abundances of phosphatidylglycerols, cardiolipin, and other phospholipids were observed in untreated 786O tumor cells. A498 cells showed a lower abundance of these species, while control HEK293 cells showed the lowest abundances.

Figure 4 presents the effect of alexidine dihydrochloride-loaded nanocarriers on the cells, showcasing selected 2D ion images of extracted metabolites (Figure 4A,C) and their intensity comparisons through ROI analysis (Figure 4B,D). In the *m*/*z* region of 700–1000, associated with complex phospholipids, the treatment significantly reduced the levels of PG (20:4) (16:0)–OH (*m*/*z* 785.5024) (Figure 4A,B) and a mixture of PG (18:1) (22:4)-O and PG (22:5) (18:0)-O (*m*/*z* 837.5273) ( Figure 4C,D) in both RCC cell lines. These species were highly abundant in untreated 786O cells, which showed a marked decrease in viability at all exposure times and tested concentrations. In contrast, they were less abundant in A498 cells, which were less sensitive to the drug and present in very low levels in the control HEK293 cell line. These findings suggest the potential importance of phosphatidylglycerols in cancer progression and indicate that tumors with higher phosphatidylglycerol levels may be more susceptible to alexidine dihydrochloride treatment.

**Figure 4:**
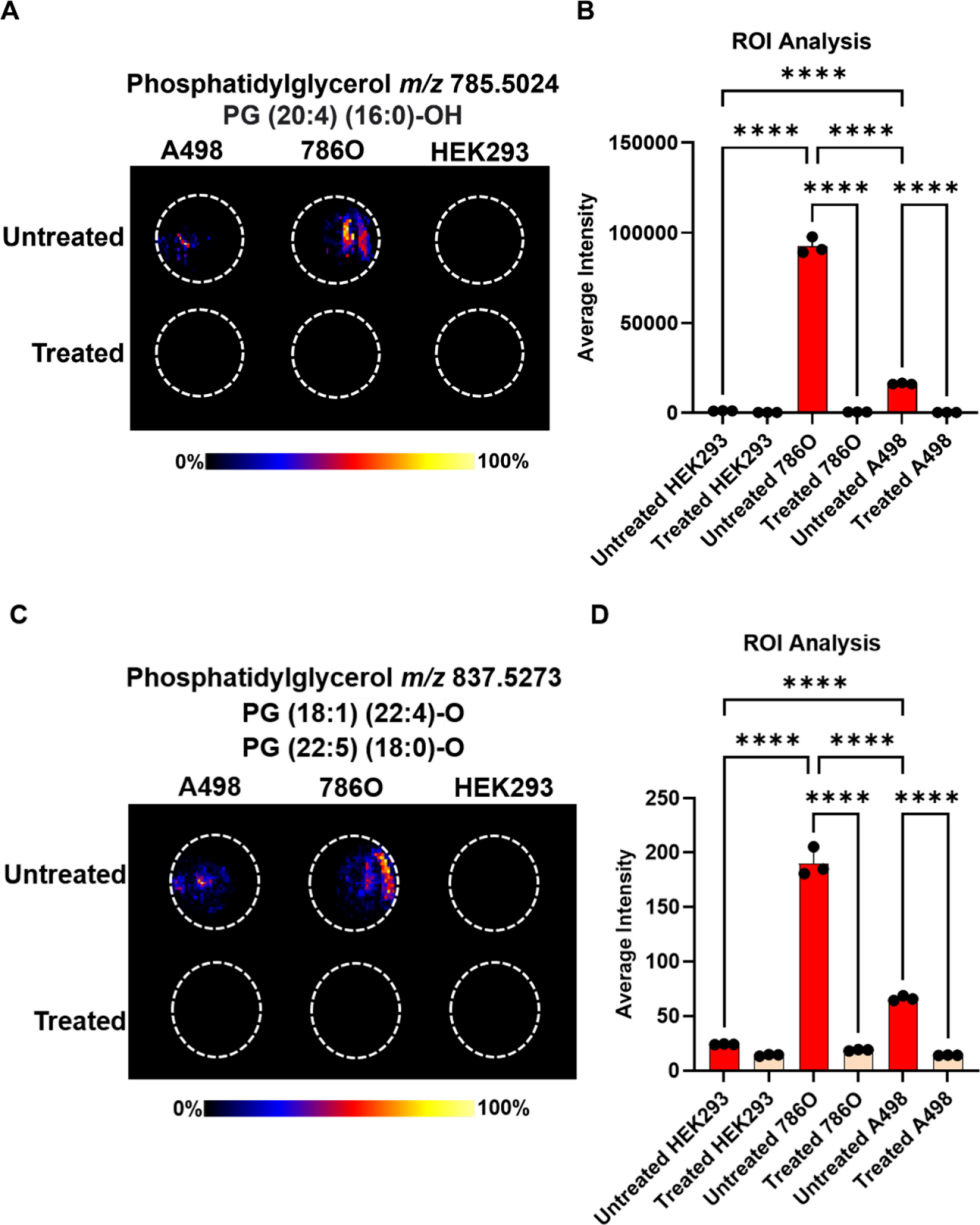
(A) DESI–MSI image of PG (20:4) (16:0)-OH in cell extracts. (B) PG (20:4) (16:0)-OH ROI analysis was performed using HDI software. (C) DESI–MSI image of PG (18:1) (22:4)-O and PG (22:5) (18:0)-O in cell extracts. (D) PG (18:1) (22:4)-O and PG (22:5) (18:0)-O ROI analyses were carried out using HDI software. Untreated HEK293 = PG intensity in untreated HEK293 cell extracts; treated HEK293 = PG intensity in treated HEK293 cell extracts; untreated 786O = PG intensity in untreated 786O cell extracts; treated 786O = PG intensity in treated 786O cell extracts; untreated A498 = PG intensity in untreated A498 cell extracts; treated A498 = PG intensity in treated A498 cell extracts. The data shown represent the average intensity of PG. Statistical analysis was performed by one-way analysis of variance with all pairwise multiple comparison procedures done by Tukey test. Symbols: ****P ≤ 0.0001.

Besides phosphatidylglycerols, elevated levels of other phospholipids, including phosphatidylcholine and phosphatidylserine, were observed in untreated tumor cells compared to untreated control cells and treated cells (Figure S5). Notably, PC (18:1) (16:0)-O (*m*/*z* 744.5900) and PS (22:5) (22:1)-O (*m*/*z* 904.5701) were highly abundant in untreated tumor cells, particularly in 786O cells. ROI analysis confirmed significantly higher levels of these phospholipids in RCC cell lines (786O and A498) than in the non-neoplastic HEK293 cell line and in untreated RCC cells compared to the treated ones. While alexidine dihydrochloride was expected to primarily affect phosphatidylglycerols through PTPMT1 inhibition, the observed decrease in other phospholipids upon treatment may be attributed to reduced demand for cell membrane components when cancer cell division is halted. Ion identities were confirmed by tandem MS using a high-mass resolution Orbitrap analyzer (Table S1 and Figures S6–S12).

### 3.4 Alexidine Dihydrochloride in Nanocarriers Inhibited Cardiolipin Synthesis

High levels of cardiolipin CL (22:5) (22:5) (22:5) (*m*/*z* 823.5070) were observed in untreated 786O tumor cells. Untreated A498 cells exhibited a significantly lower abundance of this phospholipid, though still higher than that of HEK293 cells (Figure 5A). ROI analysis revealed that cardiolipin levels were 85 times higher in untreated 786O cells and 12 times higher in untreated A498 cells than in untreated HEK293 cells (Figure 5B). Treatment with alexidine dihydrochloride-loaded nanocarriers led to a 99.8% reduction in cardiolipin in 786O and A498 cells. The higher cardiolipin levels in highly treatment-responsive 786O cells compared to untreated A498 cells suggest that tumors with upregulated phosphatidylglycerol metabolism may be particularly vulnerable to alexidine dihydrochloride treatment. The high abundance of cardiolipin in tumor cells and its marked decrease upon treatment indicate its importance in tumor progression and the effectiveness of the nanocarriers in inhibiting PTPMT1, leading to suppressed cardiolipin synthesis. Tandem MS results for cardiolipin species are shown in Figure S10.

**Figure 5:**
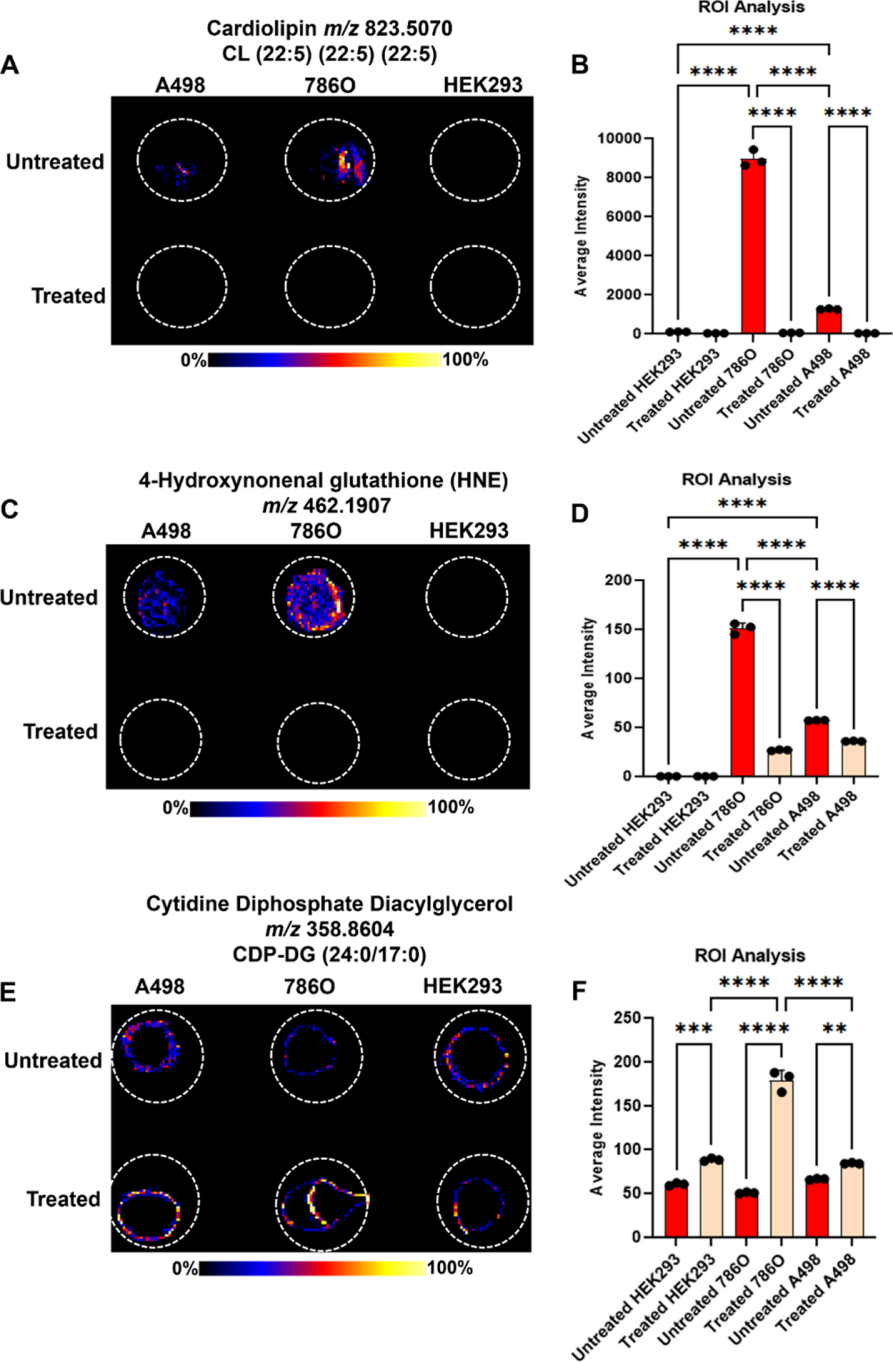
(A) DESI–MSI image of cardiolipin (22:5) (22:5) (22:5) in cell extracts. (B) Cardiolipin (22:5) (22:5) (22:5) ROI analysis. (C) DESI–MSI image of 4-HNE in cell extracts. (D) 4-HNE ROI analysis. (E) DESI–MSI image of CDP-DAG (24:0/17:0) in cell extracts. (F) CDP-DAG (24:0/17:0) ROI analysis. The data shown in ROI analysis represent the average intensity of the metabolite. Statistical analysis was performed by one-way analysis of variance with all pairwise multiple comparison procedures done by Tukey test. Symbols: **P ≤ 0.01, ***P ≤ 0.001, ****P ≤ 0.0001.

### 3.5 Alexidine Dihydrochloride in Nanocarriers Reduced 4-Hydroxynonenal Glutathione Levels, Specifically in 786O Cells

High abundances of 4-hydroxynonenal glutathione (4-HNE) (*m*/*z* 462.1907), a key metabolite involved in cellular processes like oxidative stress and cell death,^38^ were detected in untreated 786O cells compared to untreated A498 and HEK293 cells (Figure 5C). Recent studies have linked cardiolipin oxidation in mitochondria to the generation of 4-HNE.^39^ Therefore, it is reasonable to assume that alexidine dihydrochloride-loaded nanocarriers can reduce 4-HNE levels by inhibiting cardiolipin synthesis and its subsequent oxidation. Treatment with alexidine dihydrochloride in nanocarriers resulted in approximately 82% inhibition of 4-HNE in 786O cells compared to only 37% in A498 cells (Figure 5D). This suggests that high levels of 4-HNE in RCC tumors may predict their susceptibility to PTPMT1 inhibition. Notably, increased levels of HNE protein adducts have been detected in several human RCC tissues.^40^ Tandem MS results for 4-HNE are presented in Figure S11.

### 3.6 Alexidine Dihydrochloride in Nanocarriers Increased Cytidine Diphosphate Diacylglycerol Abundances in All Treated Cell Lines

The inhibition of PTPMT1 is expected to lead to the accumulation of cyclidine diphosphate diacylglycerol (CDP-DAG), an essential intermediate in cardiolipin synthesis. Higher intensities of CDP-DAG (24:0/17:0) were detected at *m*/*z* 358.8604 in all treated cells, which confirms the inhibition of PTPMT1 by the alexidine dihydrochloride-loaded nanocarriers. Furthermore, significantly higher relative abundances of this intermediate were observed in treated 786O tumor cells compared with treated A498 tumor cells and treated HEK293 cells (Figure 5E,F). These results indicate that the inhibition of PTPMT1 was more significant in 786O cells, which are apparently more metabolically active than their A498 counterparts. The accumulation of CDP-DAG with a concomitant decrease in PG observed in treated tumor cells confirms the inhibition of PTPMT1 by the alexidine dihydrochloride nanocarriers. Tandem MS results for CDP-DAG are presented in Figure S12.

## 4 Conclusions

This study demonstrates the antitumor potential of alexidine dihydrochloride-loaded nanocarriers toward human renal cell carcinoma (RCC). Alexidine dihydrochloride is a poorly water-soluble PTPMT1 inhibitor that has never been proposed for RCC treatment before. We developed a solvent-free nanocarrier system for this novel metabolic drug, achieving significant antitumor effects in highly metabolically active RCC cells. The nanocarriers not only obviated the use of organic solvents but also reduced the effective apoptotic dose of alexidine dihydrochloride and enhanced selectivity toward these RCC cells compared with non-neoplastic kidney cells. These water-dispersible unilamellar vesicles (average size: 66 ± 0 nm) exhibit high drug loading (21%), high encapsulation efficiency (99%), positive surface charge (+39.1 ± 1.3 mV), and excellent storage stability (size deviation <3% over 6 months). These properties make them promising candidates for *in vivo* RCC treatment via an enhanced EPR effect and efficient transvascular transport. Using an advanced mass spectrometry imaging method—DESI–MSI—we observed that RCC cells with high levels of certain lipids and metabolites, including phosphatidylglycerols, cardiolipins, and 4-hydroxynonenal glutathione, are particularly susceptible to treatment with alexidine dihydrochloride-loaded nanocarriers. Phosphatidylglycerols and 4-HNE are key intermediates in lipid peroxidation and the oxidative stress-related metabolic pathway, respectively. This suggests that the CDP-DAG pathway, along with lipid peroxidation processes involving 4-HNE, plays a crucial role in the progression of certain kidney tumors, and their inhibition may restrain tumor growth. Treatment with alexidine dihydrochloride-loaded nanocarriers significantly altered the CDP-DAG pathway-related metabolites, while increasing their corresponding precursors. This highlights the potential use of these molecules as biomarkers for identifying RCC tumors that may respond favorably to this novel therapeutic approach.

## Supplementary Material



## Notes

The following figures are included in the Supporting Information file: Encapsulation efficiency; stability evaluation of nanocarriers after six months of storing at 4 °C; effect of alexidine dihydrochloride in solution on cell viability; effect of alexidine dihydrochloride-loaded nanocarriers on cell viability; alexidine dihydrochloride effect on other phospholipids metabolism; Tandem MS results; and high mass resolution and tandem mass spectrometry data used for identification of molecular ions (PDF)

## Abbreviations

ANOVA: analysis of variance
CL: cardiolipin
Cryo-TEM: cryogenic transmission electron microscopy
ccRCC: clear cell renal cell carcinoma
CDP-DAG: cytidine diphosphate diacylglycerol
DDW: double-distilled water
DESI–MSI: desorption electrospray ionization mass spectrometry imaging
DLS: dynamic light scattering
DMEM: Dulbecco’s modified eagle medium
DMSO: dimethyl sulfoxide
EPR: enhanced permeability and retention
FBS: fetal bovine serum
4-HNE: 4-hydroxynonenal
MeOH: methanol
MLVs: multilamellar vesicles
MTT: thiazolyl blue tetrazolium
PBS: phosphate buffer saline
PC: phosphatidylcholine
PDI: polydispersity index
PG: phosphatidylglycerol
PS: phosphatidylserine
PTPMT1: protein-tyrosine phosphatase mitochondrial 1 enzyme
RCC: renal cell carcinoma
UV–vis: ultraviolet–visible spectroscopy
RPMI: 1640 - Roswell Park Memorial Institute medium
ROI: region of interest
ULVs: unilamellar vesicles
