# Supplementary material for: Antitumor Activity of Alexidine Dihydrochloride Nanocarriers in Renal Cell Carcinoma
Source: Mol Pharm. 2025 Sep 2;22(10):6014–24. doi: 10.1021/acs.molpharmaceut.5c00651 (PMC12505252; doi:10.1021/acs.molpharmaceut.5c00651)
Supplement: Supplementary file 1 [file mp5c00651_si_001.pdf]

Antitumor Activity of Alexidine Dihydrochloride Nanocarriers in Renal Cell Carcinoma

Adan Sultan[1], Amani Zoabi[1], Anna Morshin[1], Ori Shalev[2] Philip Lazarovici[3], Katherine Margulis[1] *

[1] Drug Delivery & Mass Spectrometry Imaging Laboratory, The Institute for Drug Research, School of Pharmacy, Faculty of Medicine, The Hebrew University of Jerusalem, Jerusalem 9112001, Israel, and the Harvey M. Krueger Family Center for Nanoscience and Nanotechnology, The Hebrew University of Jerusalem, Jerusalem 9190401, Israel.

[2]Metabolomics Center, Core Research Facility, Faculty of Medicine, The Hebrew University of Jerusalem, Jerusalem,9112001, Israel.

[3]The Institute for Drug Research, School of Pharmacy, Faculty of Medicine, The Hebrew University of Jerusalem, Jerusalem 9112001, Israel.

Results

Encapsulation efficiency

The encapsulation efficiency of alexidine dihydrochloride was determined with UV-vis by measuring the absorbance at 237 nm and calculating the percentage of alexidine dihydrochloride that is entrapped in the nanocarriers. A calibration curve for alexidine dihydrochloride in absolute ethanol was prepared for this purpose (**Figure S1**).

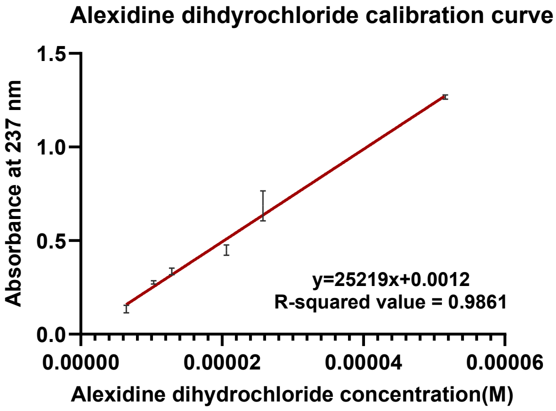

Figure S1. Calibration curve of alexidine dihydrochloride UV absorbance. Absorbance values were obtained by dilutions of alexidine dihydrochloride solutions in ethanol absolute at different concentrations. The equation for the trendline is Y= 25219x+0.0012 (R^2= 0.9861).

Stability evaluation of nanocarriers after six months of storing at 4°C

The variability in size of nanocarrier systems over time must be considered when developing drug delivery systems. Particle size and size distribution upon storage are essential factors for evaluating the stability of a colloidal dosage form. The sizes of alexidine dihydrochloride-loaded and unloaded nanocarriers were measured after six months to assess the stability of the systems. Both loaded and unloaded nanocarrier systems showed similar sizes upon storage at 4°C. The alexidine dihydrochloride-loaded nanocarriers system was more stable. It showed a much more similar size; the initial size after preparation was 66 ± 0 nm (PDI of 0.24 ± 0.009), and the size upon storage was measured at 68 ± 1 nm (PDI of 0.24 ± 0.003) **(Figure S2A).** The unloaded nanocarrier system's initial size was 87± 1 nm (PDI of 0.37 ± 0.004) and showed similar sizes of 93 ± 1 nm (PDI of 0.4 ± 0.019) upon storage **(Figure S2B).**

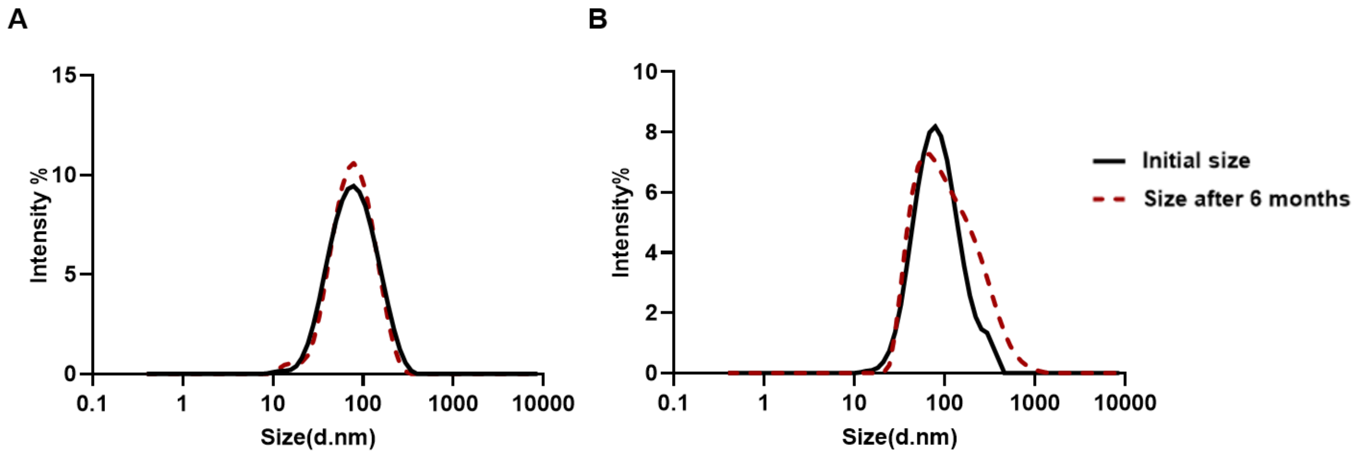

Figure S2. (A) The size of alexidine dihydrochloride -loaded nanocarriers immediately after synthesis (solid graph) and after six months (dashed graph). **(B)** The size of unloaded nanocarriers immediately after synthesis (solid graph) and after six months (dashed graph).

Effect of alexidine dihydrochloride in solution on cell viability

The impact of alexidine dihydrochloride (without nanocarriers) on the viability of 786O and HEK293 cell lines was evaluated using the MTT assay. Both tumor and normal cells were treated with alexidine dihydrochloride dissolved in 0.1%(v/v) DMSO for 72 h at 10 μM and 20 μM concentrations. 0.1%(v/v) DMSO was tested as a vehicle control (**Figure S3**). The activity of alexidine dihydrochloride was evident in both cell lines, however, we observed inconsistencies in cellular responses to the drug, with lower concentrations

resulting in greater cytotoxicity. This phenomenon can be attributed to the poor aqueous solubility of alexidine dihydrochloride, which may lead to precipitation when higher concentrations in an organic solution are mixed with aqueous cell media. These findings underscore the necessity of developing solvent-free nanocarriers to effectively assess the antitumor potential of alexidine dihydrochloride.

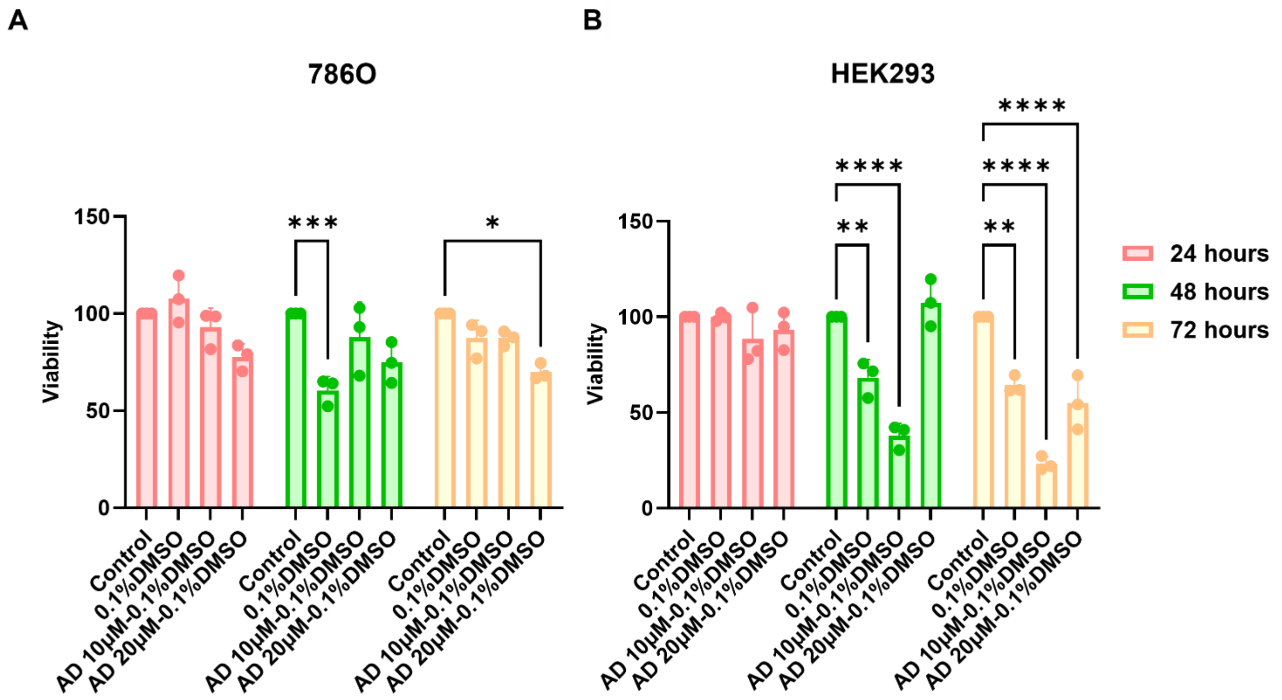

Figure S3. Cell viability analysis using MTT assay. **(A)** 786O cell viability. **(B)** HEK293 cell viability. Control=untreated cells; 0.1%DMSO= cells treated with 0.1% DMSO; AD 10 μM-0.1%DMSO = cells treated with 10 μM alexidine dihydrochloride in 0.1% DMSO; AD 20 μM -0.1%DMSO = cells treated with 20 μM alexidine dihydrochloride in 0.1% DMSO. The data shown represent three separate experiments, and the values are given as mean ± SD. Statistical analysis was performed by two-way analysis of variance with all pairwise multiple comparison procedures done by Tukey test. Symbols: * P≤0.05 **P≤0.01, ***P≤0.001, ****P≤0.0001.

Effect of alexidine dihydrochloride-loaded nanocarriers on cell viability

The effect of nanocarriers on cell viability was assessed using the MTT assay. 786O and HEK293 cells were exposed to alexidine dihydrochloride-loaded nanocarriers for 72 h at drug concentrations of 25 μM and 50 μM without organic solvent. Untreated cells were tested as a negative control, and unloaded nanocarriers were tested as a vehicle control. The data indicated that alexidine dihydrochloride-loaded nanocarriers exhibited a significantly higher and more consistent antitumor effect compared to alexidine dihydrochloride dissolved in 0.1%(v/v) DMSO. A notable decrease in cell viability was observed in RCC cells (786O) as well as in the control cells (HEK293) across all time

points and concentrations (**Figure S4**). Based on these findings, we opted to reduce the concentration of alexidine dihydrochloride to mitigate toxicity to the control cells and achieve a more targeted treatment.

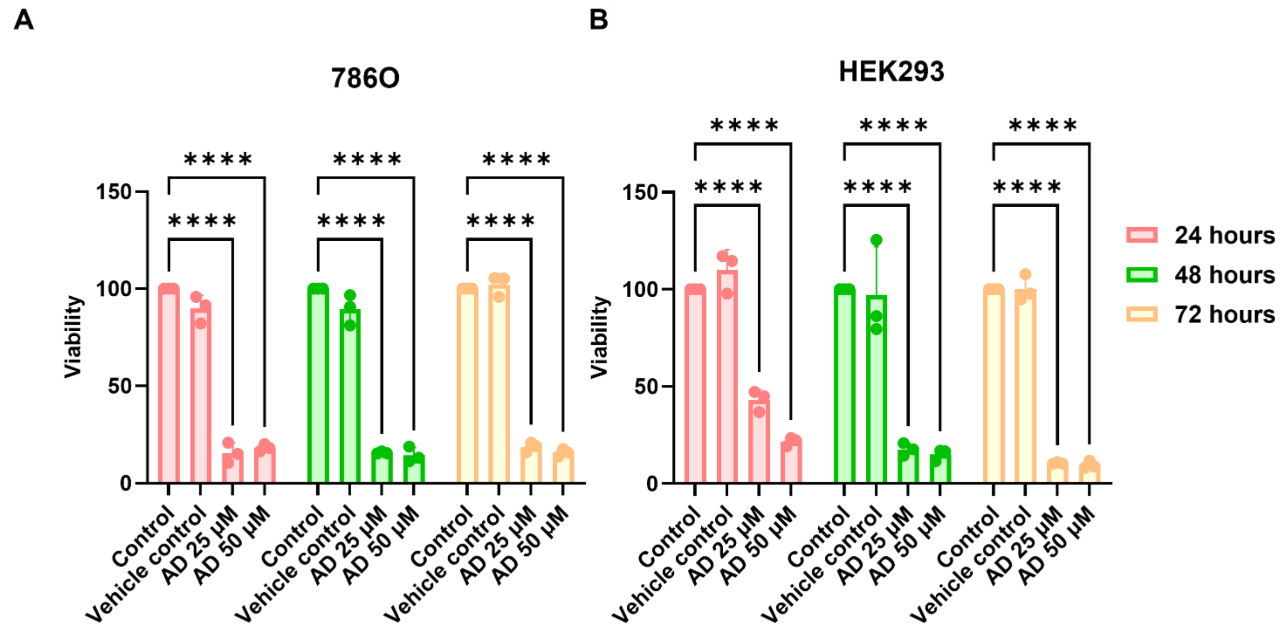

Figure S4. Cell viability analysis using MTT assay. **(A)**786O cell viability. **(B)** HEK293 cell viability. Control= untreated cells; vehicle control= cells treated with empty nanocarriers; AD 25 μM=cells treated with 25 μM alexidine dihydrochloride incorporated in nanocarriers; AD 50 μM= cells treated with 50 μM alexidine dihydrochloride incorporated in nanocarriers. The data shown represent three separate experiments, and the values are given as mean ± SD. Statistical analysis was performed by two-way analysis of variance with all pairwise multiple comparison procedures done by Tukey test. Symbols: ****P≤0.0001.

Alexidine dihydrochloride in nanocarriers affected other phospholipids' metabolism

In the *m/z* region 700-1000, where most complex phospholipids are observed, higher relative and total abundances of other phospholipids, i.e., phosphatidylcholine and phosphatidylserine, were detected in the untreated tumor cells (**Figure S5**). These metabolites were more abundant in the 786O cells, which showed a significant decrease in cell viability after being exposed to alexidine dihydrochloride-loaded nanocarriers over 72 h. Based on the ROI analysis derived from HDI processed DESI-MSI data, the levels of the phospholipids were found to be higher in RCC cell lines 786O and A498 as compared to non-neoplastic cell line HEK293 (**Figure S5B and S5D**). Additionally, treatment with

alexidine dihydrochloride nanocarriers resulted in a significant decrease in these phospholipids in all cell lines.

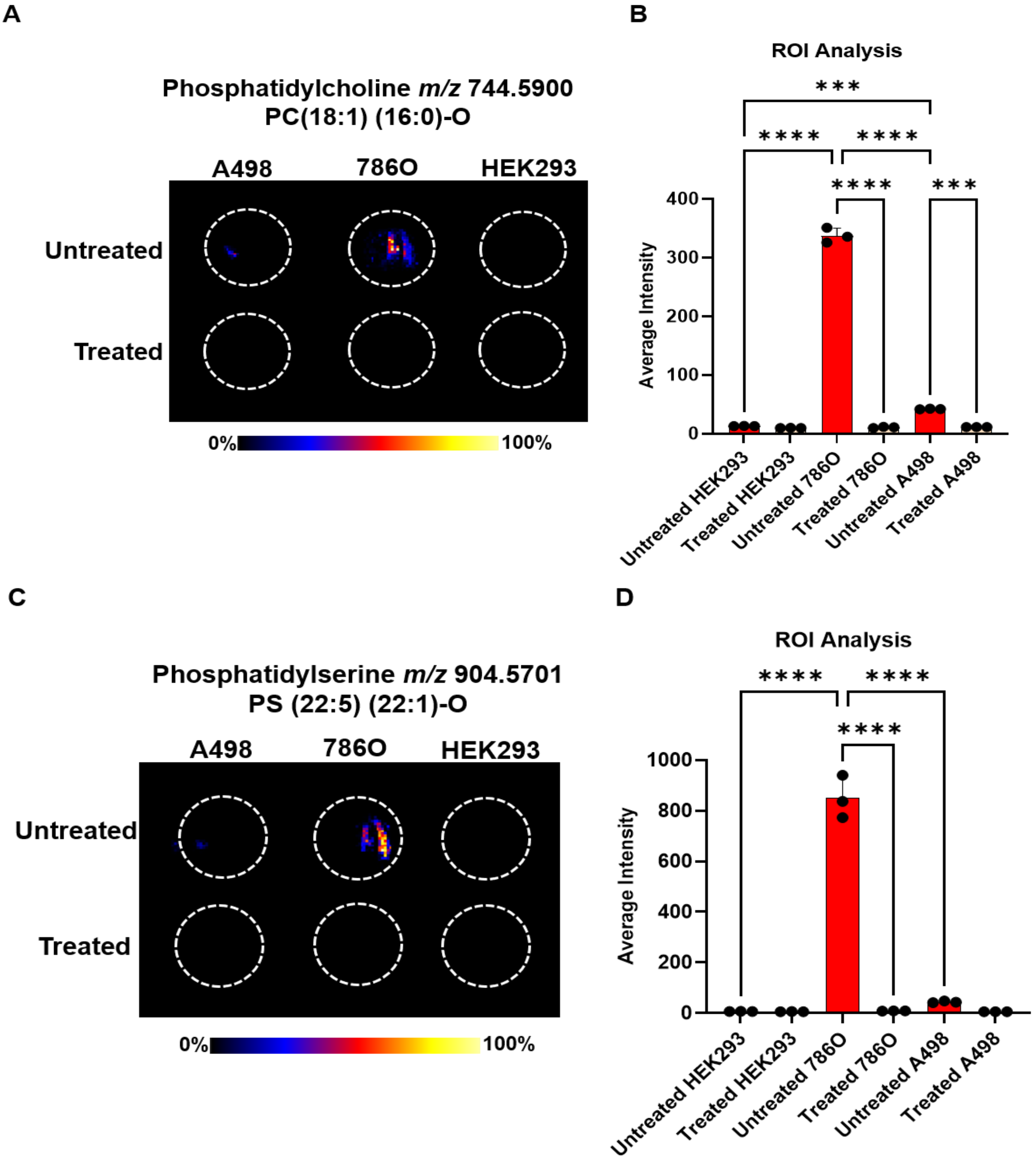

Figure S5. (A) DESI-MSI image of PC (18:1) (16:0)-O in cell extracts. **(B)** PC (18:1) (16:0)-O ROI analysis. **(C)** DESI-MSI image of PS (22:5) (22:1)-O in cell extracts. **(D** PS (22:5) (22:1)-O ROI analysis. Untreated HEK293= intensity in untreated HEK293 cell extracts; treated HEK293= intensity in treated HEK293 cell extracts; untreated 786O- intensity in untreated 786O cell extracts; treated 786O=intensity in treated 786O cell extracts; untreated A498= intensity in untreated A498 cell extracts; treated A498=intensity in treated A498 cells extracts. The data shown represents the average intensity of the metabolite. Statistical analysis was performed by one-way analysis of variance with all pairwise multiple comparison procedures done by Tukey test. Symbols: ***P≤0.001 ****P≤0.0001.

Table S1 High mass resolution and tandem mass spectrometry data used for identification of molecular ions.

High mass resolution	Main fragment ions	Tentative ion attribution	Adduct	Exact *m/z*	Mass error (ppm)	Proposed ion formula
744.5900	281.2491,255.2324	PC (18:1) (16:0)-O	[M-H]	744.5907	-0.940114	$C_{42}H_{84}NO_7P$
823.5070	329.2498	CL (22:5) (22:5) (22:5)	[M-2H]	823.5095	-3.035788	$C_{97}H_{150}O_{17}P_2$
462.1907	113.0240	4-Hydroxynonenal glutathione	[M-H]	462.1907	0.000	$C_{19}H_{33}N_3O_8S$
358.8604	78.9588	CDP-DG (24:0/17:0)	[M-3H]	358.8620	-4.458538	$C_{53}H_{99}N_3O_{15}P_2$
904.5701	329.2482,327.2324	PS (22:5) (22:1)-O	[M-H]	904.5701	0.000	$C_{50}H_{84}NO_{11}P$
837.5273	281.2481,331.2634, 283.2637, 329.2482	PG (18:1) (22:4)-O and PG (22:5) (18:0)-O	[M-H]	837.5282	-1.074591	$C_{46}H_{79}O_{11}P$
785.5024	303.2324,255.2325	PG (20:4) (16:0)-OH	[M-H]	785.4969	7.001937	$C_{42}H_{75}O_{11}P$

Tandem MS results

The following spectra represent tandem MS at a higher energy collisional mode (HCD 10-40) of different lipids and metabolites detected in untreated and treated cell extracts.

Structure

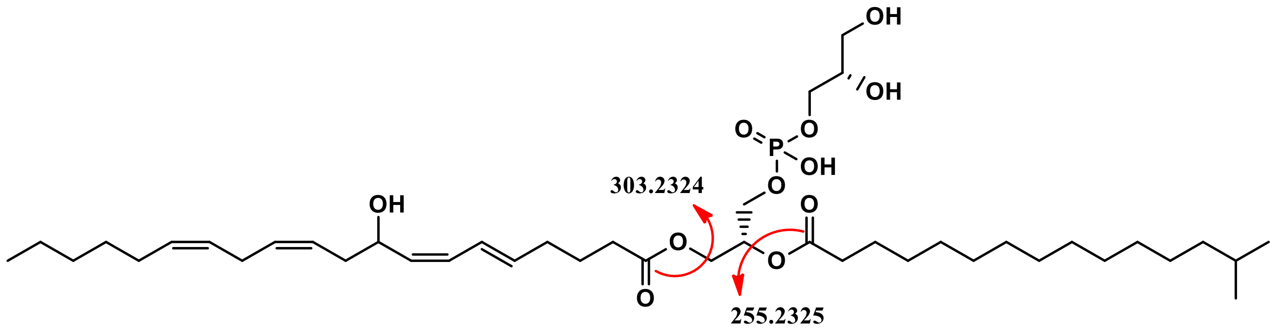

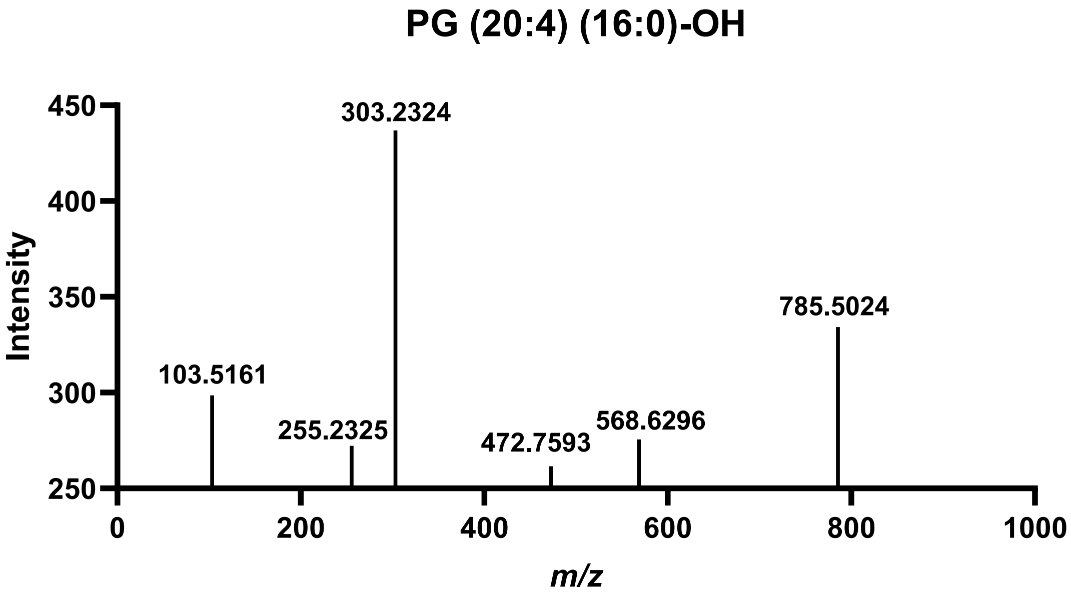

Figure S6. HCD of *m/z* 785.5024.

Structure

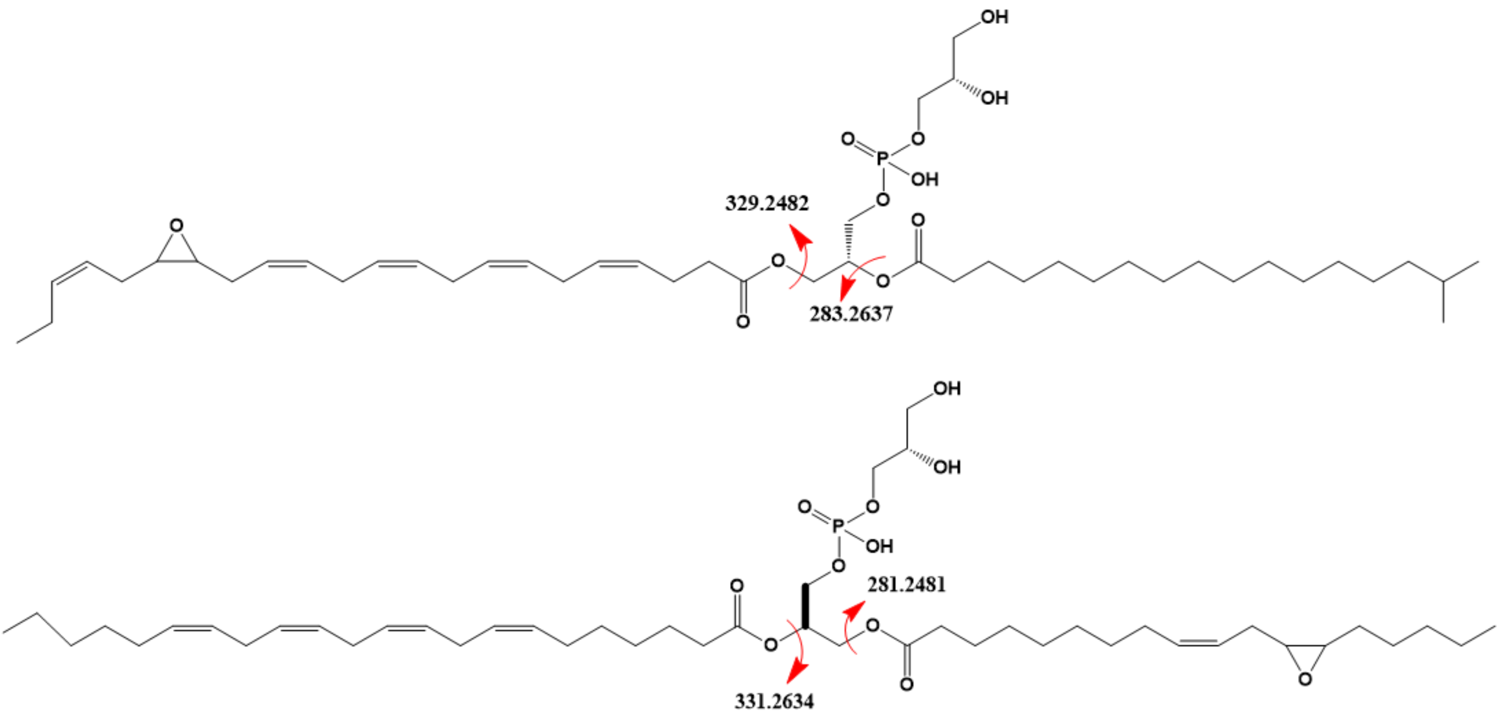

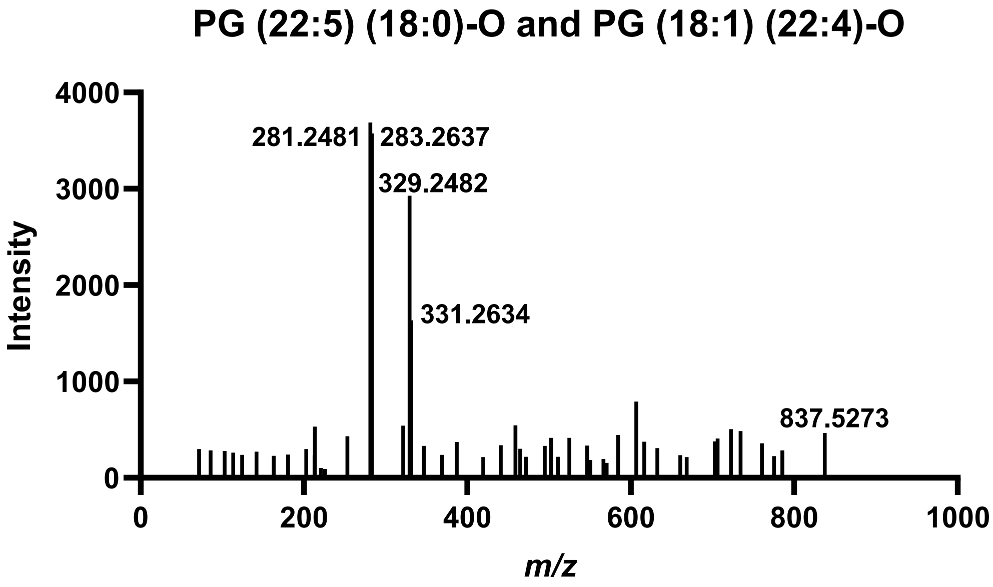

Figure S7. HCD of *m/z* 837.5273.

Structure

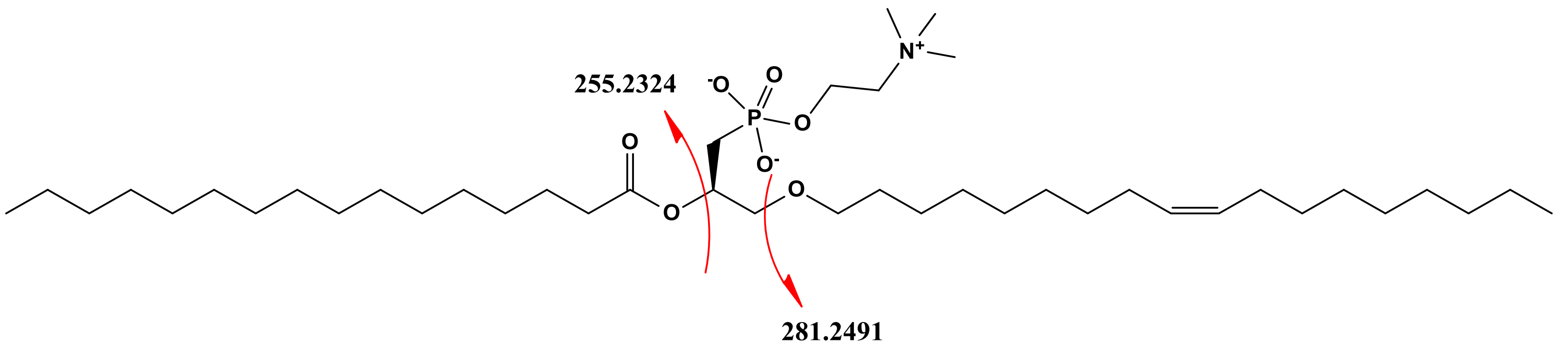

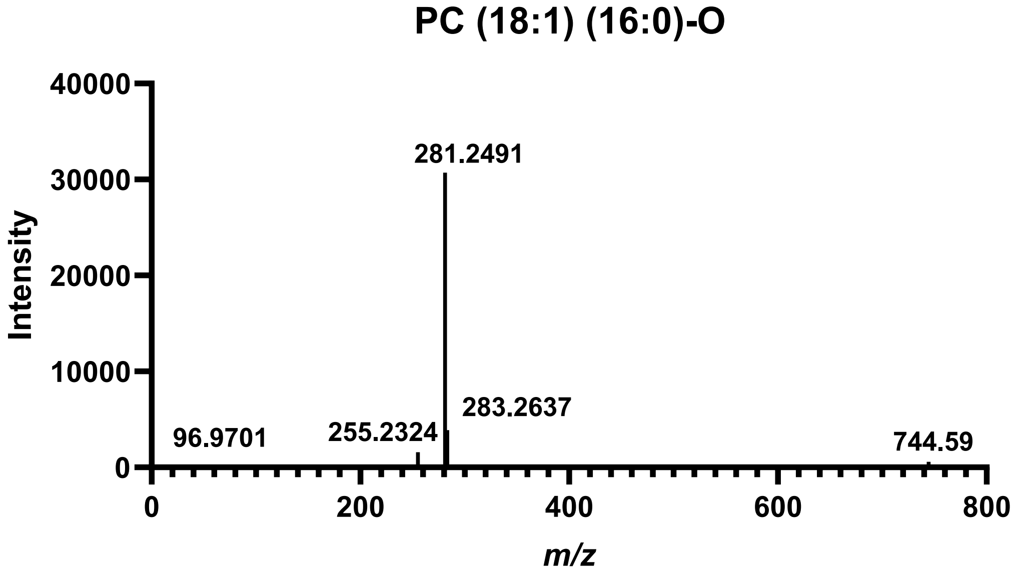

Figure S8. HCD of *m/z* 744.5900.

Structure

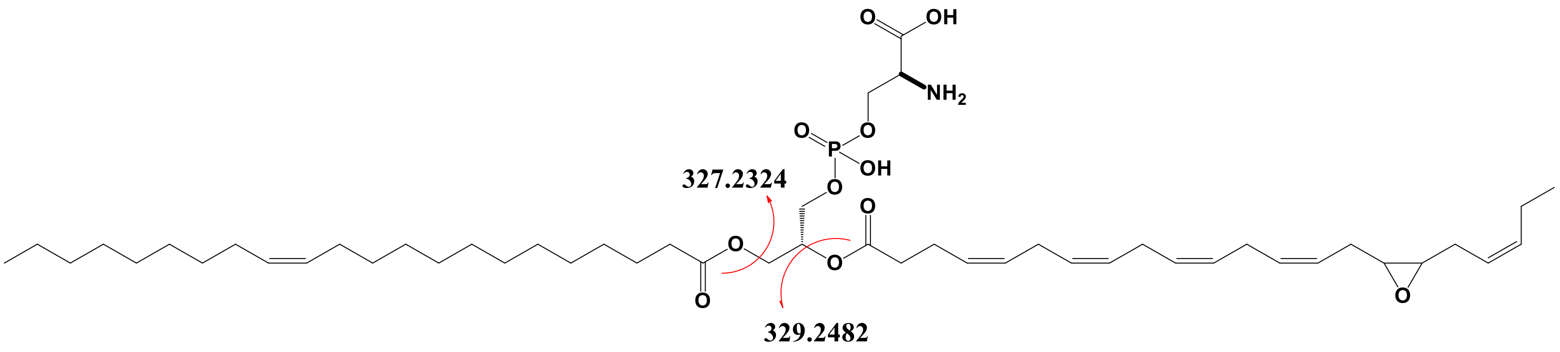

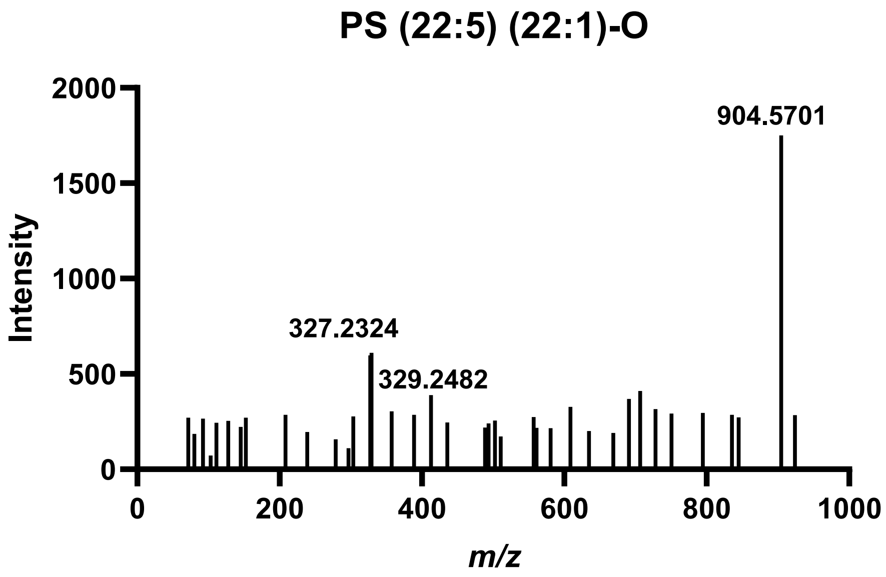

Figure S9. HCD of *m/z* 904.5701.

Structure

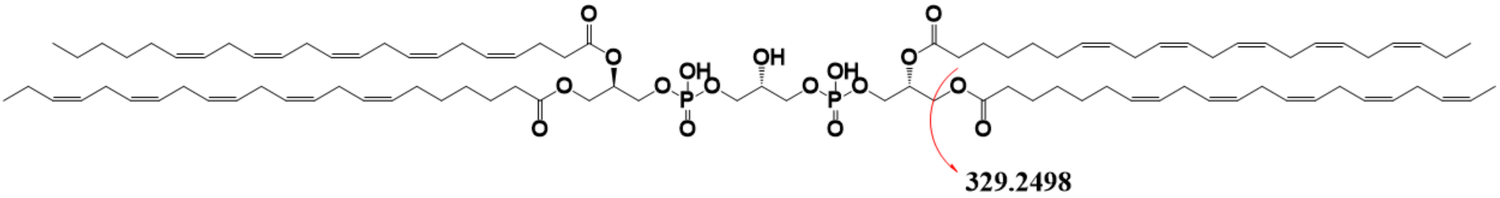

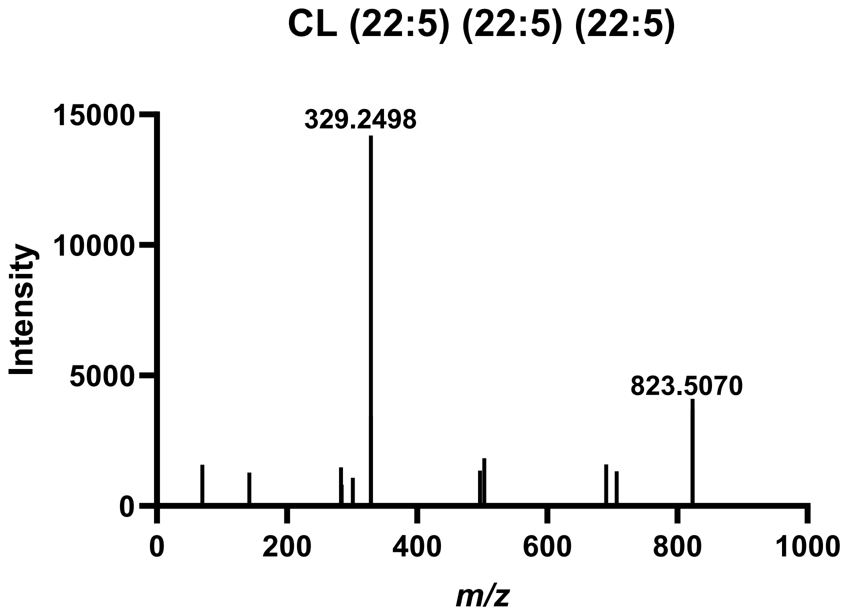

Figure S10. HCD of *m/z* 823.5070.

Structure

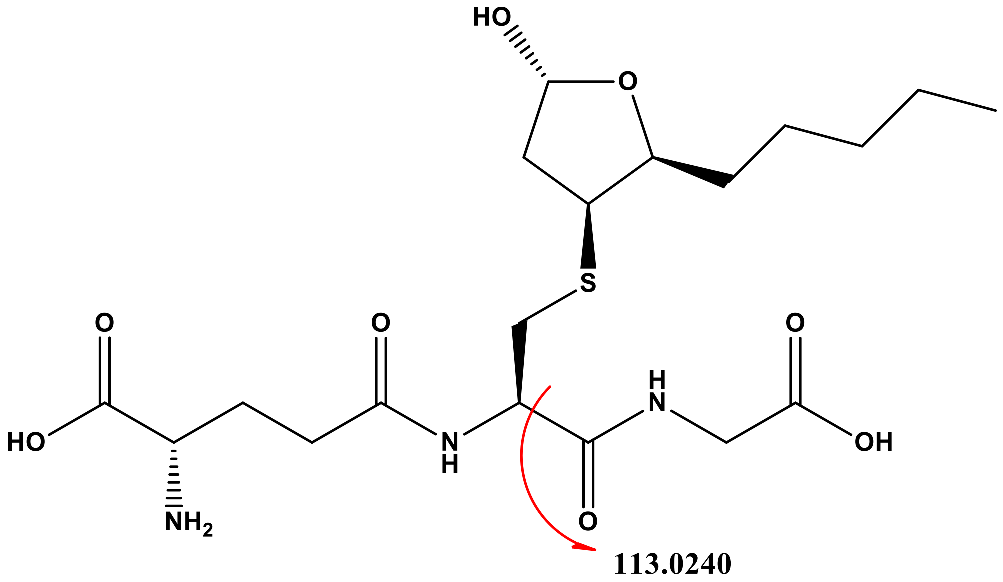

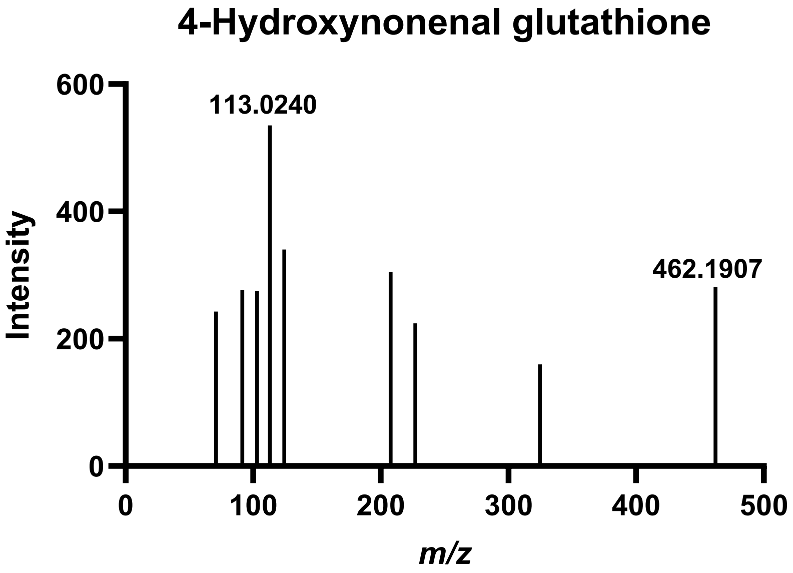

Figure S11. HCD of *m/z* 462.1907.

Structure

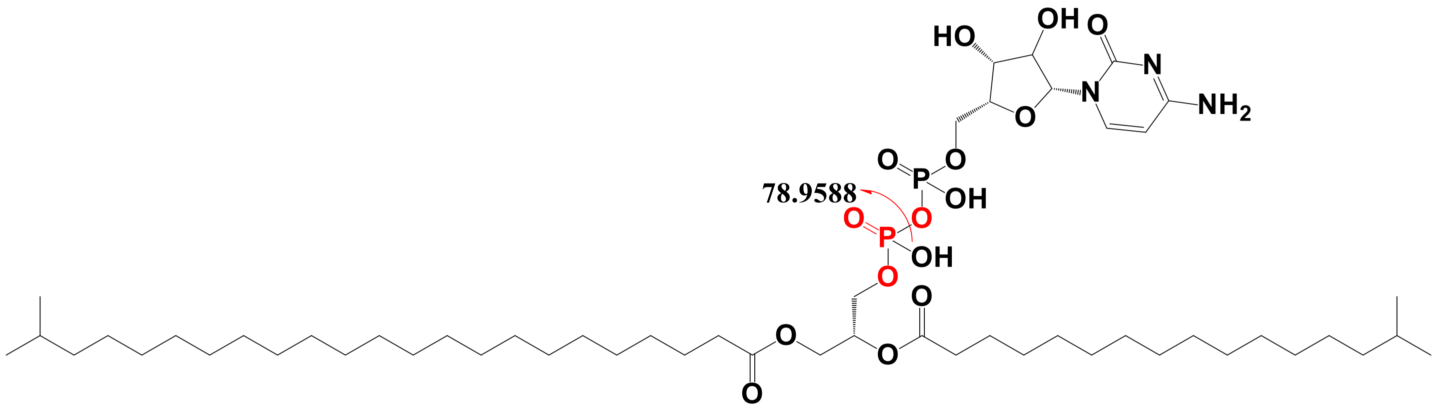

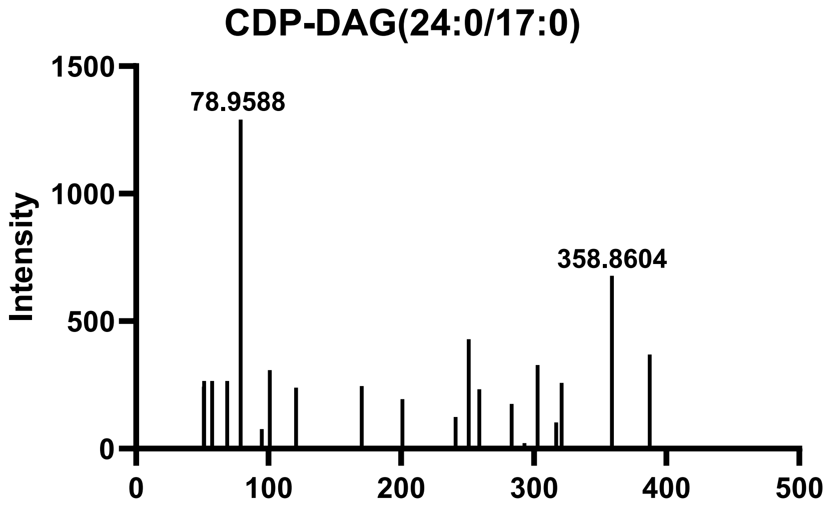

Figure S12. HCD of *m/z* 358.8604.
